# Exosomal targeting and its potential clinical application

**DOI:** 10.1007/s13346-021-01087-1

**Published:** 2022-01-01

**Authors:** Jiao He, Weihong Ren, Wei Wang, Wenyan Han, Lu Jiang, Dai Zhang, Mengqi Guo

**Affiliations:** 1 The First Clinical Medical Institute, Henan University of Chinese Medicine, Zhengzhou, Henan People’s Republic of China; 2 Department of Laboratory Medicine, The First Affiliated Hospital of Henan University of Chinese Medicine, Zhengzhou, Henan 450000 People’s Republic of China

**Keywords:** Exosomes, Engineering, Transformation, Targeting

## Abstract

Exosomes are extracellular vesicles secreted by a variety of living cells, which have a certain degree of natural targeting as nano-carriers. Almost all exosomes released by cells will eventually enter the blood circulation or be absorbed by other cells. Under the action of content sorting mechanism, some specific surface molecules can be expressed on the surface of exosomes, such as tetraspanins protein and integrin. To some extent, these specific surface molecules can fuse with specific cells, so that exosomes show specific cell natural targeting. In recent years, exosomes have become a drug delivery system with low immunogenicity, high biocompatibility and high efficacy. Nucleic acids, polypeptides, lipids, or small molecule drugs with therapeutic function are organically loaded into exosomes, and then transported to specific types of cells or tissues in vivo, especially tumor tissues, to achieve targeting drug delivery. The natural targeting of exosome has been found and recognized in some studies, but there are still many challenges in effective clinical treatments. The use of the natural targeting of exosomes alone is incapable of accurately transporting the goods loaded to specific sites. Besides, the natural targeting of exosomes is still an open question in disease targeting and efficient gene/chemotherapy combined therapy. Engineering transformation and modification on exosomes can optimize its natural targeting and deliver the goods to a specific location, providing wide use in clinical treatment. This review summarizes the research progress of exosomal natural targeting and transformation strategy of obtained targeting after transformation. The mechanism of natural targeting and obtained targeting after transformation are also reviewed. The potential value of exosomal targeting in clinical application is also discussed.

**Graphical abstract:**

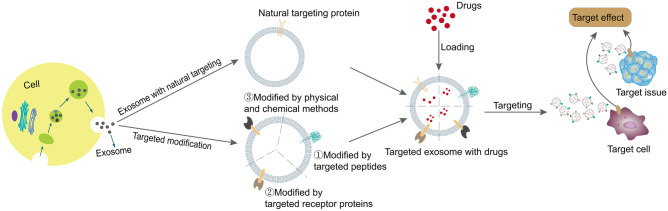

## Background

**Fig. 1:**
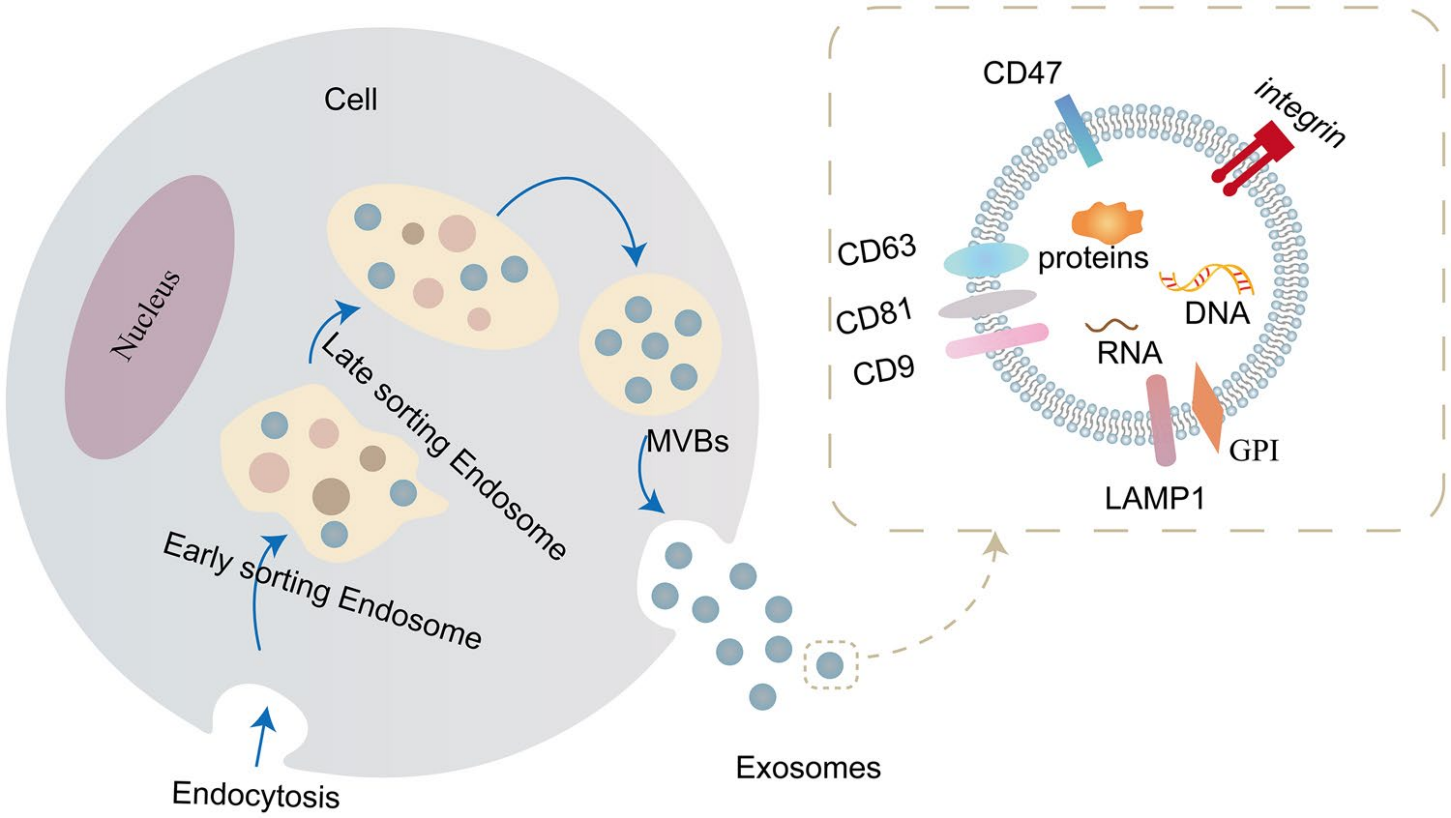
Exosomal biogenesis

In multicellular organisms, intercellular communication plays an important role in regulating biological processes and maintaining homeostasis [1]. Extracellular vesicles (EVs), as carriers of cell membrane with intracellular proteins, lipids, and nucleic acid, are important ways of intercellular communication [2–4]. Exosomes are a subgroup of EVs, and their production processes include two invagination of plasma membrane and the formation of multivesicular bodies (MVBs) containing intracellular vesicles (ILVs), which subsequently fuse with the plasma membrane and eventually secrete exosomes into the extracellular matrix through exocytosis [5–7] (Fig. 1). Due to the characteristics of exosomes, their contents are easy to transmit macromolecular signals through cell membranes in the form of biological activity, so they can be used as saclike carriers for intercellular transport of macromolecules such as nucleic acids and proteins [6]. Studies on the properties and functions of exosomes have shown that exosomes can participate in a variety of pathological and physiological processes, including central nervous system diseases, myocardial ischemia/circulatory injury, liver and kidney injury, angiogenesis, and so on [8]. The research on exosomes as nanocarriers can provide key information for the research progress of different diseases.

Due to the body’s own physiological barrier and immune defense function against foreign bodies, drugs and other active substances will be prevented from reaching specific sites in the process of disease treatment. Therefore, the treatment of disease requires weak immunogenicity, low toxicity, and targeting delivery carrier through physiological barrier to complete drug delivery. EVs can transport small molecules (such as chemotherapeutic drugs, etc.), proteins, small interfering RNA (siRNA), and microRNAs (miRNA), which can potentially avoid lysosomal degradation to achieve cargo transport at the cellular level [9]. In recent years, EVs have become new therapeutic carriers in immunotherapy, regenerative medicine and drug delivery [10]. Simultaneously, exosomes with low immunogenicity, good biodegradability, and have stronger protective effect on biological activity goods, and can effectively through the body's physiological barrier (such as blood–brain barrier, etc.) or escape immune recognition [11–13], the characteristics of exosomes as the targeting delivery of drugs’ and other bioactive molecules' carriers have attracted the interest of the researchers.

Some special membrane proteins (such as tetraspanins and integrins) are expressed on the surface of EVs, which makes EVs with naturally targeting to cells or organs [9, 14]. However, recent studies have shown that after intravenous injection of unmodified EVs, EVs can accumulate rapidly in organs of the reticuloendothelial system (such as liver and spleen), but only a small amount of EVs are delivered to the brain after systemic administration, while the volume of target modified EVs in the brain is significantly higher than that of unmodified EVs [15]. Therefore, the natural targeting of exosomes alone is not enough to recruit them to the specific sites, so it is necessary to modify exosomes to complete a more accurate targeting delivery process. Some studies have found that the surface modification of exosomes can achieve targeting site-specific drug delivery, which will become a new field in drug delivery research. In this paper, the natural targeting ability of exosomes was discussed, and the strategy and mechanism of engineering targeting modification of exosomes were reviewed. Furthermore, the potential value of exosome targeting in clinical application was discussed.

Exosomal biogenesis is formed by the invagination of the cell membrane to form intracellular multivesicular bodies (MVBs), which is matured after fusion with endoplasmic reticulum and Golgi vesicles and protein classification mechanism, and the mature MVBs are fused with the plasma membrane and secreted to extracellular. Exosomes are a highly heterogeneous population, which have the ability to induce complex biological reactions. There are tetraspanins, integrins, and surface adhesion proteins attached to their membranes, and rich in nucleic acids, proteins, lipids, and other bioactive substances.

## Exosomes

Exosomes are vesicles about 30–150 nm in diameter, which can be secreted by a variety of cells [16], including immune cells [17], cancer cells [18], and stem cells [19] and so on. They are distributed in almost all body fluids, including blood [20], urine [21], tears [22], semen [23], cerebrospinal fluid [24], and so on. Exosomal generation process involves the formation of ILVs and the fusion of MVBs with plasma membrane [25]. After fusing with plasma membrane, the mature MVB’s release is interfered by a variety of pathways; meanwhile, the secreted exosomes can be absorbed and internalized again, and release their contents to the receptor cells, which plays the function of information transmission between cells [26]. Exosomes are rich in a variety of bioactive substances, including mRNAs, miRNAs, proteins, lipids and other contents, as well as CD63, CD9, and many other membrane protein signal molecules [27]. Its biological formation and contents’ sorting are mainly related to endosomal sorting complex required for transport (ESCRT) and independent ESCRT pathway for the transport of cargos [28–30]. Through their surface molecules’ receptor-ligand interaction, endocytosis, or direct fusion with the targeting cell membrane, EVs can release contents into the cytosol of target cells, thus achieve the targeting transportation of cargos [31]. Studies have shown that normal and cancer-related fibroblasts can affect adjacent or distant cells through miRNAs derived from exosomes, which in turn affects the migration, invasion, and metastasis of cancer cells, and induces tumor drug resistance [32].

Exosomes are the important medium of information transmission between cells and play an important role in the occurrence and development of many diseases [33]. Compared with the traditional liquid biopsy, due to the protective effect of exosomal lipid bilayer membrane, the information from the mother cell can exist stably for a long time and maintain a certain biological activity [34]. Exosome is rich lots of proteins and nucleic acids, especially RNAs such as messenger RNA (mRNA), long non-coding RNA (lncRNA), and circular RNA (circRNA), which is easier to reveal the molecular mechanism of disease development [35]. Therefore, exosomes, as the potential biomarker, are of great value in clinical diagnosis [36, 37]. The separation method of exosomes should be technically simple, not to damage the structural integrity of exosomes, and be conducive to drug loading and clinical applications. At present, the methods of separation, extraction, and enrichment of exosomes mainly include: ultracentrifugation [38, 39], ultrafiltration [40, 41], kit extraction method (ExoQuickTM method, etc.) [42], magnetic bead immunoassay [39], size exclusion chromatography [43], microfluidic technology [44], etc. Exosomes are heterogeneous, and their particle diameters are different. At present, there is no unified standard for the separation and purification of exosomes. The advantages and disadvantages of various separation methods are summarized in Table 1.

**Table1:** Comparison of advantages and disadvantages of exosomal extraction methods

Separation methods	Principle	Advantages	Disadvantages
Ultracentrifugation [38]	Separation based on molecular density, size and shape	Low cost, suitable for a large number of samples and low pollution risk [39]	Time consuming, low yield, high cost, easy to damage integrity, and biological activity
Ultrafiltration [41]	Separation according to molecular size and shape	High speed, no special equipment, suitable for large samples	Not easy to distinguish components of similar size
Kit extraction method [42]	Biological macromolecules are precipitated by using high molecular hydrophobic polymers	Suitable for large samples [45]	High price and cost
Magnetic bead immunoassay [46]	The immobilized antibody selectively binds to the antibody receptor on the exosome membrane to realize the separation	Well specificity [46]	Not applicable to large samples
Size exclusion chromatography [43]	Separation according to molecular size and size	Separation according to the particle size, high yield, and can ensure the integrity and biological activity of exosomes [47]	Other methods shall be combined, and multiple samples cannot be operated at the same time
Microfluidic technology [48]	According to the characteristics of exosomes, specific devices are used for separation	High speed, low cost, simple operation and easy automation	High material and technical requirements, high cost, not suitable for large samples

Because exosomes have the ability to transfer RNAs, proteins and other bioactive substances, which can play a crucial role in a variety of biological processes, such as angiogenesis, antigen presentation, apoptosis, immune response, and intercellular signal transduction, and furtherly affect the physiological and pathological processes of many diseases [49], including tumors [50–52], neurodegenerative diseases [53–55], infection [56], and other diseases. In the treatment of diseases, it is an ideal drug delivery system to ensure the bioavailability of drug delivery and reduce the toxicity of drugs at the same time.

Exosomes can be used as natural carriers of drug delivery, meanwhile, making it a targeting capability by genetic engineering and chemical modification, which can enhance the affinity to target cells or organs, reduce the damage of drugs to normal tissues and cells, and finally reduce the toxicity and side effects of drugs [57, 58]. The technology of drug loaded by exosomes can be divided into two categories: one is to load drugs by directly treating the exosomes themselves; the other is to load the exosomes secreted by the parent cells with drugs [39]. The treatment methods mainly include electroporation method [59], chemical transfection method [60, 61], CO incubation method [62], etc. Some people also load drugs into exosomes by means of saponin penetration, freeze–thaw cycle, ultrasonic treatment, or extrusion [63]. As a drug carrier, exosomes are used to deliver therapeutic substances to tumor cells efficiently and specifically, which provides a new idea and practical strategy for tumor treatment.

### Natural targeting exosomes

Exosomes are secreted by cells, existing in almost all biological body fluids, and are very attractive in tracking the occurrence and development of diseases and immunotherapy. Among the known types of cells that produce exosomes, exosomes derived from human mesenchymal stem cells are easy to obtain and have certain therapeutic effects [64]. It has been found that exosomes derived from bone marrow mesenchymal stem cells used in the treatment of graft-versus-host disease have well tolerance of repeated injection and have no substantial side effects, which may provide a potential new safety tool for the treatment of refractory graft-versus-host disease and other potential inflammation-related diseases [65].

The biological distribution and accumulation of exosomes from different cells are different, and this natural tendency can induce off-target effect of exosomes during drug delivery. Delivery of drugs by exosomes in a specific and controlled manner is a key step in increasing therapeutic effectiveness and reducing off-target effects. Proteomic evidence indicates that some membrane proteins can be specifically expressed on the surface of tumor associated exosomes, which will become attractive targets for clinical diagnosis or treatment. To a certain extent, some specific surface molecules that expressed on the surface of exosomes secreted by different cells can make exosomes have natural targeting. For example, tumor metastasis is not random, some cancer cells will be targeted to attach to specific organs, tumor cell-derived exosomes play a key role in the targeting metastasis of cancer. Cancer cells can release corresponding exosomes to find target organs and connect tumors with the microenvironment of target organs. The targeting exosomes contain some oncogenes and oncoproteins, resulting in targeting metastasis of tumors. At the same time, this phenomenon can inspire us to use exosomes for tumor targeting therapy. Examples of clinical application of natural targeting exosomes are summarized in Table 2.

**Table 2:** Clinical application of natural targeting exosomes

Targeting membrane protein	Targeting ligand/ Target cells	Function	Reference
Integrin α4β7	Hepatitis E virus endothelial cells	Inhibit the migration of gut-tropic T lymphocytes to the intestine	[69]
Integrin α3β1	The inner cyclic nine peptide LXY30	Distinguish between cancer and non-cancer associated exosomes, and reduced the uptake of exosomes from SKOV-3 parent cells	[67]
Integrin αvβ5	Kupfer cells	Promote tumor metastasis to the liver	[71]
Integrin α6β4 and Integrin α6β1	The fibroblasts and epithelial cells of the lungs	Promote tumor metastasis to the lung	[71]

#### Exosomal membrane protein integrin induces its targeting

Integrin is composed of α and β subunits and participates in a variety of physiological and pathological biological processes, including inflammation, thrombosis, cell adhesion, and migration [66–68]. Integrins can also be expressed on the surface of exosomes derived from cancer cells that overexpress integrins. T cell adhesion molecule integrin not only regulates the tissue-specific homing pattern of cancer cells and immune cells, but also regulates the specific enrichment state of exosomes derived from these cells [69]. Exosome-mediated immune cell homing microenvironment remodeling can affect immune cell migration and host defense, as well as tumor metastasis, thus becoming a potential therapeutic target.

**Fig. 2:**
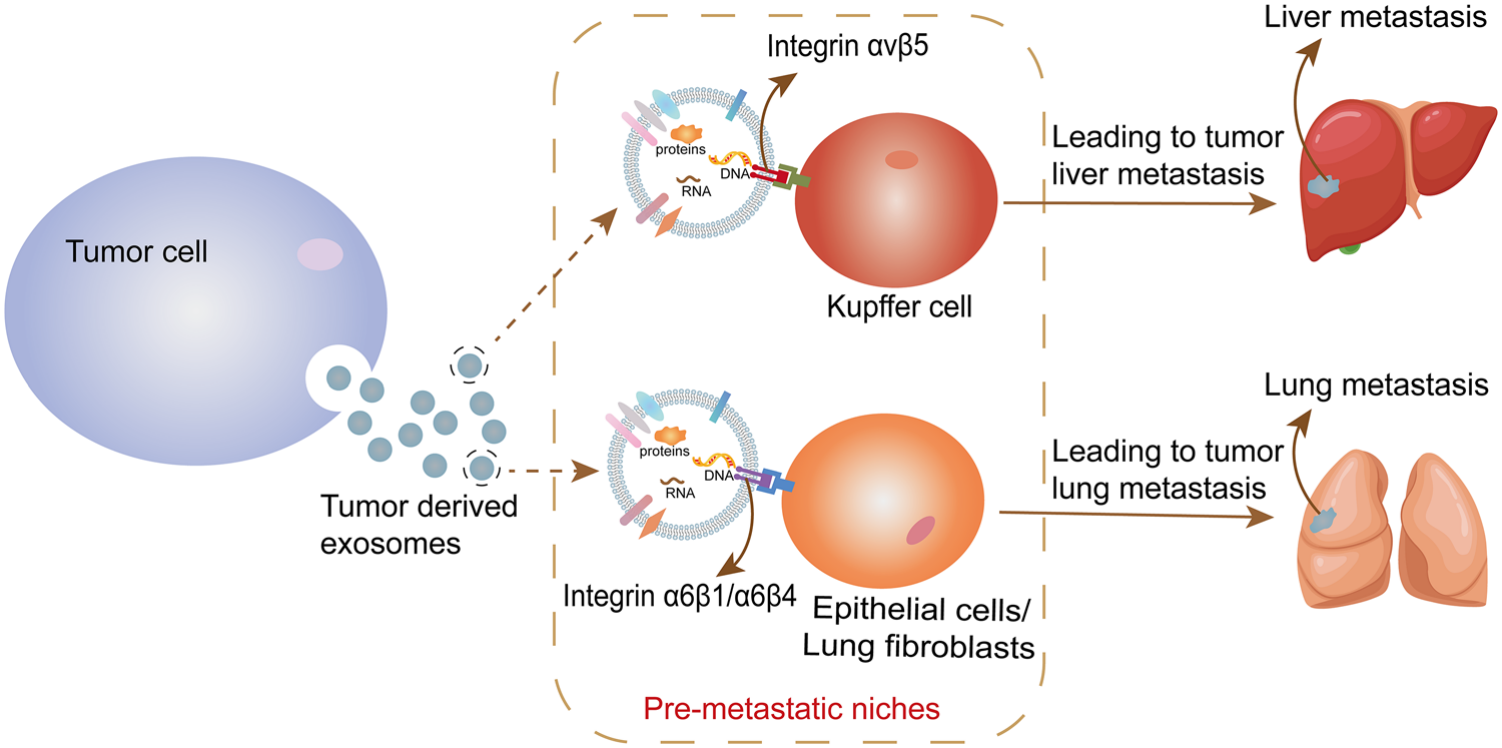
Exosomal membrane protein integrin induces its targeting metastasis

Some studies have found that T cell-derived exosome expressing integrin α 4 β 7 can preferentially target intestinal hepatitis E virus endothelial cells and change the expression of microenvironment tissue, so as to inhibit the migration of enterophil T lymphocytes to the intestine [70]. Both SKOV-3 ovarian tumor cells and their exosomes can express integrin α3β1, which can be specifically targeted by the inner cyclic nine peptide LXY30. LXY30 not only showed high specificity and affinity for exosomes expressing integrin α3β1, but also reduced the uptake of exosomes from SKOV-3 parent cells. This study improves the potential application of exosomes in nanomedicine therapy and diagnostic systems [68]. By participating in the formation of the niche before metastasis and the remodeling of the tumor microenvironment at the primary site, the integrin expressed on exosomal surface can promote the organ-specific targeting metastasis of the tumor [69]. Exosomes expressing integrin αvβ5 can specifically bind to Kupffer cells, which contributed to metastasis spreading toward the liver [71]. Exosomes expressing integrin α6β4 and α6β1 can regulate metastasis spreading to the lungs by combining with fibroblasts and epithelial cells, which suggests that organ-specific metastasis can be limited by inhibiting the expression of exosomal integrin or inhibiting the binding of integrin to target cells to some extent [71] (Fig. 2). This study reveals that exosomes have great potential in early diagnosis, targeting drug delivery and disease treatment. We can further study the targeting mechanism of exosomes and apply it to targeting treatment of diseases in the future.

Exosome integrin expression may serve as a potential predictor of organ-specific metastasis in patients. Exosomes secreted by breast cancer cells express specific integrin membrane proteins that mediate the remodeling of immune microenvironment and induce tumor-specific metastasis. Exosomes expressing integrin αvβ5 bind specifically to Kupfer cells to promote metastasis to the liver, while exosomes expressing integrin α6β4 and integrin α6β1 can bind to fibroblasts and epithelial cells in the lung to regulate metastasis to the lung.

#### Targeting of other exosomes membrane proteins

Remarkably, the expression of EVs’ surface membrane proteins can promote the interaction and fusion between membranes [72], and realize the targeting transport of the contents loaded by exosomes [73]. Exosomal membrane is mainly composed of Lamp, glucose phosphate isomerase (GPI), and four transmembrane proteins (such as CD63, CD9, CD81, CD47, etc.) [2], which can fuse with the receptor on the surface of the corresponding receptor cell, thus affecting the specific uptake of the receptor cells [74, 75]. Among them, CD47 is an integrin-related transmembrane protein, part of its function is to protect cells from phagocytosis [76, 77]. CD47 is the ligand of signal regulatory protein alpha (SIRPα), CD47-SIRPα binding will send out “don’t eat me” signal [78, 79], which can correspondingly increase exosomal half-life period in circulation [80]. Oncogenic Ras has been proved to endow pancreatic cancer cells with strong macropinocytosis, which may promote the uptake of exosomes by cancer cells [81]. The presence of CD47 on exosome helps to escape the role of immune clearance of host in circulation. Meanwhile, RAS mediated megacytosis enhances the ability of pancreatic cancer cells to take out exosomes. The results show that this characteristic of exosomes enhances their ability to transfer RNA interference (RNAi) to specific target cancer KRAS in pancreatic cancer [80]. Morelli et al. [82] found that milk fat globule (MFG)-E8/lactic acid adherent protein, CD11a, CD54, phosphatidyl serine, four transmembrane proteins, and αv/β3 on the surface of bone marrow dendritic cells could induce exosomes to target receptor cells and extracellular matrix proteins. Hood et al. [83] have respectively injected melanoma cell-derived exosomes and liposomes with the same treatment into mice. It was found that exosomes in mice were mainly located in the lymph nodes near the injection site, while the liposomes with the same treatment were uniformly distributed in the distant lymph nodes, and exosomes derived from melanoma cells could target hematopoietic stromal cells and promote neovascularization. Therefore, compared with liposomes of similar size, tumor-derived exosomes have significant targeting selectivity for the location of lesions in vivo, and this specific homing effect is closely related to exosomal membrane proteins. The instability and low systemic bioavailability of curcumin (cur) are one of the main obstacles in the clinical application of cur in the treatment of cancer and other inflammation related diseases. In order to use nanoparticle exosomes as cur delivery carriers, the researchers mixed cur with exosomes at 22 °C, and then carried out sucrose gradient centrifugation. It was found that the incorporation of cur into exosomes could improve the solubility, stability, and bioavailability of cur to improve its pharmacokinetics [62]. In order to further determine the specific targeting of cur carrying exosomes (cur-exosomes) to organs, the biological distribution of fluorescent dye labeled exosomes in mice was observed by fluorescence imaging technique. Within one hour after injection, fluorescent signals were mainly detected in mouse liver, lung, kidney, and spleen. Flow cytometry analysis showed that cur-exosomes not only targeting CD11b^+^ Gr-1^+^ cells in peripheral circulation, but also enhanced/increased the transmission of cur to CD11b^+^ Gr-1^+^ cells [62]. CD11b^+^ Gr-1^+^ cells are one of the main cell populations related to the pathogenesis of disease. Exosome-induced cur targeting CD11b^+^ Gr-1^+^ cells may provide a novel treatment for inflammation-related diseases and even cancer. It can be seen that exosomes have great potential in disease diagnosis, targeting drug delivery, and potential disease treatment. We can study the targeting delivery ability of exosomes and apply it to the treatment of a variety of diseases.

In one study of encephalitis diseases, it is found that cur wrapped in exosomes can be quickly transported to the brain and significantly reduce the tumor volume. This may be because exosomes promote the stability of drugs in vivo and enhance the effect of chemotherapeutic drugs. In order to further determine the specific targeting of exosomes to microglia, the exosomes were labeled with fluorescent dye PKH26 to observe their biological distribution in vivo. After intranasal delivery of exosomes for 15 min, double fluorescent PKH26-positive cells were found in brain microglia. Within 1 h after injection, more than 60% of microglia were PKH26 positive; it shows that the injected exosomes can target deliver drugs to microglia [84]. Through this study, we can deliver drugs encapsulated by exosomes to the brain through intranasal administration, which may improve the direct delivery of drugs to the central nervous system, and this method has certain targeting specificity and advantages of noninvasive administration. However, because microglia are not the only target cells of exosomes, the biological effects of other cells, especially immune cells infiltrating the brain, including NK cells or CD11bGr-1 myeloid cells, need to be further studied. Meanwhile, the study found that unmodified exosomes are quickly cleared after they are recognized by the mononuclear phagocyte system, which limits their accumulation in related tissues to a great extent [85]. In addition, the transmembrane efficiency of unmodified natural exosomes is low, which is not enough for clinical treatment [86]. In order to further improve the targeting gathering ability of the exosomes in the process of drug transport and reduce the toxicity to normal cells during treatment, exosomes can be engineered to give it specific targeting to cells or organs.

#### Targeting exosomes constructed by engineering

Studies have proved the therapeutic potential of exosomes in animal models of various diseases, and most reports mainly focus on cancer treatment. Cancer metastasis is the leading cause of cancer-related death worldwide, and new tumor treatment strategies are needed. Although exosomes play an important role in disease diagnosis and treatment, their limited effectiveness still needs to be considered. Because the exosomes released by most cells are complex and have limited targeting to specific cells, improving stability and targeting ability are two important problems in manufacturing engineering exosomes [87]. Targeting specific cancer cells is an important prerequisite for the application of exosomes in cancer therapy. In order to produce exosomes target cancer cells, a variety of engineering methods have been explored. There are many strategies to obtain engineering targeting exosomes, such as targeting modification of exosomes by targeting peptides or receptor protein. Compared with biological ligands, homing peptides or targeting protein fragments can bind to small molecules on the surface of exosomes more easily and effectively, and they can show highly specific interaction with targeting proteins [88]. Examples of exosomes modified with targeting peptides and receptor proteins as drug delivery systems are summarized in Table 3.

**Table 3:** Engineering exosomes as drug delivery systems

Targeting peptide/protein	Receptor	Target cells/organs	Function	Reference
IRGD peptide	Lamp2b	Breast cancer cell	Targeting delivery of DOX and effectively inhibit tumor growth	[94]
CSTSMLKAC peptide	Lamp2b	Ischemic myocardium	Reduce inflammation, apoptosis and fibrosis, enhance angiogenesis, and cardiac function	[95]
c (RgdyK) peptide	Integrin αvβ3	Ischemic brain injury area	Targeting delivery of cur and inhibits the inflammatory response in lesion area	[98]
RGE peptide	Neurokinin-1	Glioma	Targeting delivery of cur	[101]
c-Met binding peptide	c-Met	TNBC cells	Targeting delivery of DOX	[102]
GE11 peptide	EGFR	Breast cancer cell	Targeting delivery of the tumor inhibitory miRNA	[110]
RVG peptide	Lamp2b	Brain neurons, microglia and oligodendrocytes	Targeting delivery of siRNA and knockdown of Alzheimer’s disease related genes	[112]
RVG peptide	Acetylcholine receptor	Neuron cell	Targeting delivery opioid receptor mu siRNA to treat morphine addiction	[113]
Apo-A1	SR-B1 receptor	Liver cancer cells	Targeting delivery Functional miR-26a	[118]

#### Exosomes modified by targeting peptides

**Fig. 3:**
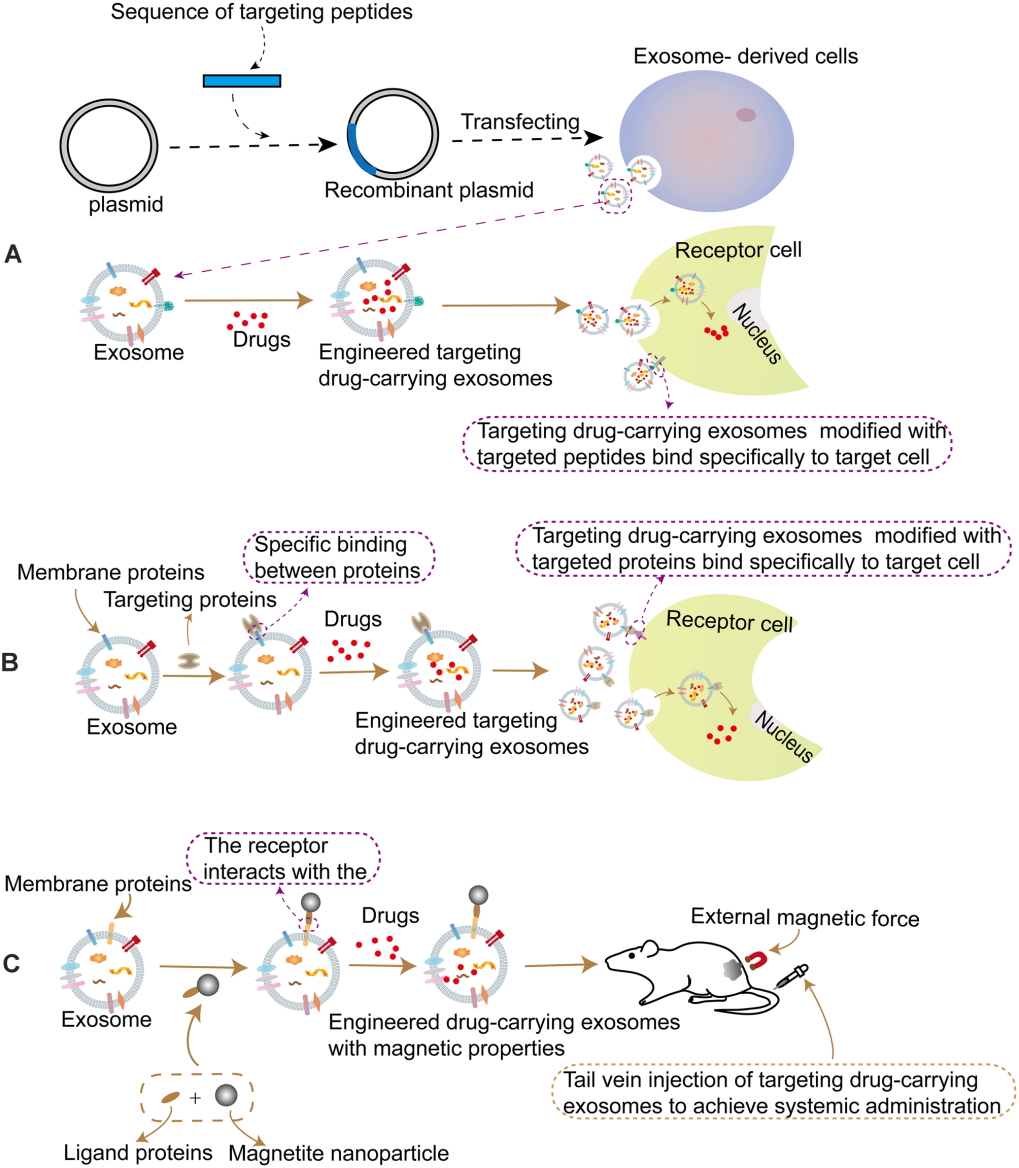
Engineered targeting exosomes as drug carriers for disease treatment. **A** In order to optimize the targeting of exosomes and enhance their specific targeting, we modified exosomes with targeting peptides. By constructing recombinant plasmids carrying targeting peptides, the obtained fusion proteins are transfected into cells and exosomes modified with targeting peptides are obtained, so that exosomes have stronger tissue or cell specificity, and therapeutic drugs are loaded into exosomes to achieve targeting and specific treatment of diseases. **B** Targeting modification of exosomes using the target protein. The targeting proteins are bound to the exosome membrane proteins. The drugs are loaded into exosomes, and the targeting delivery of drugs at specific sites is completed through the specific binding of target proteins to target cells. **C** Targeting exosomes with magnetic nanoparticles. Superparamagnetic nanoparticles are combined with exosomes to make exosomes magnetic, and drugs are loaded into exosomes with magnetic properties. Exosomes carrying drugs are enriched at specific sites under the action of external magnetic fields to achieve targeting delivery of drugs

Due to the weak targeting of exosomes, targeting peptides are fused with exosome surface molecules by using engineering modification technologies (such as molecular cloning, lentivirus packaging technology, biological orthogonal chemistry, etc.) to construct fusion peptides to realize the targeting transformation of exosomes. Finally, the drugs are loaded into the modified exosomes, and the drugs are targeted and recruited to specific sites through the transport of exosomes, so as to realize the specific treatment of diseases (Fig. 3A).

Polypeptides are considered to be the best choice for targeting modification ligands because of their small size, low immunogenicity, and specific binding ability with proteins and antigens [89, 90]. One way to improve the specificity of exosomes to target cells is to add peptide ligands fused with exosome membrane proteins. Modification of exosomal surface with known cyclic peptide IRGD (CRGDKGPDC) can enhance the permeability of antineoplastic drugs to blood vessels and tumor tissues, further enhance the efficacy of anticancer drugs, and reduce the side effects of drugs on normal tissues [91–93]. TIAN Y et al. [94] fused the IRGD peptide targeting integrin αν to the characteristic membrane protein lysome-associated membrane glycoprotein 2B (lamp2b) of exosomes derived from immature dendritic cells (imDCs), and then transferred the chemotherapeutic drug doxorubicin (DOX) into modified exosomes by electrical stimulation to obtain DOX-loaded IRGD exosomes (IRGD-exos-DOX). In the tumor nude mouse model experiment, IRGD-exos-DOX and blank-exos-DOX without IRGD were fluorescently labeled and injected into mice. The results showed that the fluorescence signal of IRGD-exos was detected at the tumor site and reached the peak about 2 h after injection. The strongest fluorescence was detected in the liver 2 h after injection of blank-exos, but no specific fluorescence was observed at the tumor site at any time. Moreover, compared with the control group, no significant tissue damage was observed in the IRGD-exos-DOX treatment group [94]. Target secretory bodies have high affinity for integrin αν-positive breast cancer cells in vivo and in vitro. Systemic administration of this DOX delivery system significantly inhibited tumor growth without causing significant toxicity [94]. Thus, the modified target exosomes are safe and effective drug delivery carriers for targeting tumor therapy. In addition, some researchers used molecular cloning and lentivirus packaging technology to fuse the membrane protein Lamp2b rich in exosomes with ischemic myocardial targeting peptide CSTSMLKAC (IMTP) to prepare ischemic myocardial targeting exosomes (IMTP-exosomes). In vitro experiments showed that IMTP-exosomes could be internalized more effectively by hypoxia damaged H9C2 cells than blank-exosomes. IMTP-exosomes can specifically target ischemic myocardium, so as to improve the targeting therapeutic effect of exosomes on ischemic myocardium [95]. Cyclopeptide (c (RgdyK)) has high affinity with integrin αvβ3 on the surface of vascular endothelial cells after cerebral ischemia [96, 97]. The researchers used a simple, rapidly and biologically orthogonal chemical method to integrate c (RgdyK) into exosomal surface, while loading the drug onto the engineered exosomes, c (RGDyK)-coupled exosomes (cRGD-Exo) can target ischemic brain injury [98]. cRGD-Exo is considered to be the targeting delivery carrier of drugs for the treatment of cerebral ischemia because it can inhibit the inflammatory reaction and apoptosis in the lesion area more effectively than using drugs alone [98]. Because the RGE peptide can specifically bind to Neurokinin-1 [99, 100], neuropilin-1-targeting peptide (RGERPPR), superparamagnetic iron oxide nanoparticles (SPIONs), and cur are loaded on the exosomes (RGE-Exo-SPION/cur) by electroporation and click chemical methods, thus the engineered targeting exosomes with imaging and therapeutic functions can be obtained, and these engineered exosomes can successfully pass through the blood–brain barrier [101]. It provides good results for targeting imaging and treatment of gliomas [101]. At the same time, the tumor nude mouse model experiment found that the imaging signal of RGE-Exo-SPION/cur at the lesion site was stronger than that of un-targeting SPION group and non SPION group. It can identify glioma in the early stage with clear boundary and shape.

In order to further improve the tumor targeting of exosomes, polypeptides were used to modify the surface of macrophage derived exosomes to target the overexpressed mesenchymal-epithelial transforming factor (c-Met) in triple-negative breast cancer (TNBC) cells, and the polylactic acid-glycolic acid nanoparticles loaded with DOX were packaged into the modified exosomes to realize the preparation of tumor targeting drug delivery exosomes, which can improve the tumor targeting, cell uptake efficiency, and anti-tumor activity of macrophage-derived exosomes, as well as the anti-tumor efficiency of chemotherapeutic drugs, and provide a new way for effective targeting therapy of TNBC [102]. Nucleic acid drugs have therapeutic potential to a certain extent, but their clinical application is limited by the lack of appropriate drug delivery carriers. Epidermal growth factor receptor (EGFR) is associated with the progression of a variety of cancers, including breast cancer [103], lung cancer [104, 105], cervical cancer [106], and colorectal cancer [107]. The increased expression of EGFR in some epithelial tumors can be used as a receptor target for cancer drug delivery system [108]. GE11 is a potentially safe and effective targeting part, which can specifically bind to EGFR and be used in EGFR-mediated selective drug delivery system [109]. Exosomal donor cells were engineered to secrete GE11 peptide modified exosomes (GE11-exosomes), and the tumor inhibitory miRNA was transferred to breast cancer tissue with high expression of EGFR by exosomes [110]. The data show that the number of GE11-exosomes reaching the tumor is three times higher than that without GE11 peptide, which reveals that GE11-exosomes have good tumor targeting in vivo [110]; this may provide a platform for miRNA replacement therapy for various cancers. Effective delivery of therapeutic functional molecules in vivo requires the binding of exosomes to specific receptors. Relevant studies to explore the expression strategies of exosome targeting modified peptides may be particularly useful for the transformation of promising treatment strategies based on exosomes from preclinical research to human treatment.

#### Exosomes modified by targeting receptor proteins

The study of targeting editing of exosomes has gradually become a hot spot in the application of exosomes in disease treatment. In the targeting modification technique, the specific targeted receptor proteins bind to exosome surface proteins, so as to realize the targeting enrichment of the exosomes to the receptor cell [111] (Fig. 3B).

Recent studies have explored a new method for targeting delivery of therapeutic RNA to cancer cells. By editing the dendritic mother cell-derived exosomes, rabies virus receptor glycoprotein (RVG) polypeptides that can target and bind to the lamp2b membrane protein of nerve cells were made on the membrane surface. The exogenous siRNA was introduced into RVG modified exosomes (RVG-exosomes) by electroporation and injected intravenously into mice; it was found that exogenous siRNA can be specifically transported to neurons, microglia, and oligodendrocytes in the brain, so as to achieve exosome targeting drug delivery [112]. Liu et al. modified exosomes with neuron-specific RVG peptides, which can bind to acetylcholine receptors expressed on neuronal cells, so that the RVG-exosomes can effectively cross the blood–brain barrier (BBB). Opioid receptor µ (MOR) siRNA was effectively packaged into exosomes. The data results showed that RVG peptide modification on exosome membrane specifically induced exosomes to target receptor cells with RVG peptide, so as to effectively deliver mor siRNA to receptor cells to regulate mor gene expression [113]. In conclusion, the study revealed that RVG-exosomes delivered siRNA to the central nervous system through BBB to regulate gene expression, while natural exosomes without RVG modification could not deliver siRNA to the central nervous system or regulate target gene expression. Targeting peptides fused to the N-terminal of exosome related transmembrane protein lamp2b may be degraded during exosome biogenesis. In order to inhibit the degradation of peptides, researchers developed a new exosome modified by RVG peptide lamp2b fusion protein containing glycosylation motif. The introduction of this glycosylation motif not only protects the polypeptide from degradation, but also induces the increase of the overall expression of lamp2b fusion protein. In addition, glycosylated stable peptide enhanced the targeting delivery of exosomes to neuroblastoma cells, indicating that this glycosylation did not eliminate peptide target interaction and significantly enhanced the targeting delivery of exosomes to neuroblastoma cells [114]. Therefore, after knowing that RVG peptide targeting neurons is expressed on the surface of exosomes, it is hoped that this drug delivery technology targeting exosomes can be used to solve brain related medical problems in the future.

The apolipoprotein Amur1 (Apo-A1), the main component of high-density lipoproteins, is a known targeting of scavenger receptor B type 1 (SR-B1) receptor [115]. SR-B1 is highly expressed on the surface of a variety of cancer cells, including HepG2 [116], melanoma cells [117], and so on. In order to obtain Apo-A1-modified exosomes, the cells that secretes exosomes were modified by fusing Apo-A1 with the transmembrane protein CD63 on the surface of T cells [118]. Functional miR-26a is loaded into Apo-A1-modified exosomes by electroporation to achieve targeting delivery of therapeutic miR-26a to cancer cells [118]. One of the main challenges of gene therapy in clinical application is to develop gene therapy vectors that spread to the brain. The specificity of targeting exosomes, the ability to load exogenous genes, the ability to systematically exert gene therapy, and the characteristics of immune evasion are useful values for the clinical application of oligonucleotides. The development of targeting of exosomes acupuncture to other tissues and the improvement of its yield and efficiency will contribute to the application of this gene transfer clinical treatment technology. Compared with normal hematopoietic cells, interleukin-3 receptor (IL3-R) is highly expressed in chronic myeloid leukemia (CML) cells, which can be used as a receptor target for tumor drug delivery system [119]. The researchers used genetic engineering technology to make human embryonic kidney (HEK) 293 T cells express protein Lamp2b, and fuse it with IL3 (IL3-Lamp2B) to obtain IL3-Lamp2B modified exosomes [111]. The protein lamp2b was expressed in HEK293T cells by genetic engineering technology, and the protein was fused with interleukin 3 (IL3), and finally the IL3-lamp2b modified exosomes were obtained. The results show that protein modified exosomes containing IL3-lamp2b and imatinib can specifically target tumor cells in vivo, deliver tumor-suppressive drugs (imatinib or BCR-ABL siRNA) to CML cells, and inhibit the growth of cancer cells in vitro and in vivo [111]. Both in vivo and in vitro data show that imatinib can be quickly delivered to CML cells using engineered exosomes, so as to overcome the systemic drug toxicity associated with long-term administration. Targeting drug delivery systems will enable drugs to be directed to specific tissues, requiring lower doses. It can be seen that engineered exosomes have great development prospects in drug targeting delivery. They can not only realize drug targeting transport, but also reduce drug toxicity and improve drug solubility in cancer treatment. At the same time, it provides a reasonable basis for overcoming pharmacological drug resistance.

#### Targeting exosomes with physical and chemical modifications

In addition to the above-mentioned engineering modification techniques of targeting exosomes, physical and chemical modification are also commonly used, such as the preparation of magnetic exosomes by coating magnetic nanoparticles, or physical and chemical editing techniques such as covalent modification of exosomes and the use of ligand/receptor interactions. Examples of physically and chemically modified exosomes for targeting drug delivery are summarized in Table 4.

**Table 4:** Physically and chemically modified exosomes for targeting drug delivery

Targeting ways/ligand	Targeting methods/receptor	Target cells/organs	Function	Reference
Exosomes-SPION	The magnetic field in tumor site	Liver cancer	Targeting delivery of DOX and effectively inhibit tumor growth	[123]
Exosomes-SPION	The magnetic field in tumor site	Melanoma	Targeting delivery of tumor necrosis factor and effectively inhibit tumor growth	[124]
SIRPα	CD47	Macrophages and tumor cells	Antagonizes the interaction between CD47 and SIRPα	[129]
AA-PEG	Sigma receptor	Lung cancer cells	Targeting delivery of PTX	[134]
Hydrophilic cholesterol coupled with RNA aptamer or folic acid	RNA ligand receptor / folate receptor	Cancer cells	Targeting delivery of siRNA and miRNA	[135]

#### Physical modification

With the development of science and technology, the technology of targeting modification of exosomes using magnetic nanoparticles has also emerged, which uses the combination of superparamagnetic nanoparticles and exosomes as carriers to complete drug gathering and targeting delivery in specific parts (Fig. 3C).

Reticulocyte (RTC), as the main source of exosomes in blood flow, releases 1014 exosomes every day during the maturation of erythrocytes, so it is a potential source of sufficient and safe exosomes [120, 121]. It is known that RTCs-derived exosomes (RTCs-exosomes) in blood contain a variety of membrane proteins, including transferrin receptors (TfR) [120, 122]. Superparamagnetic nanoparticles (SPMNs) were coated on transferrin, and then multiple SPMNs were anchored to each RTCs-exosome through the interaction between transferrin receptor and transferrin collect and purify exosomes-based superparamagnetic nanoparticle cluster (SMNC-exosomes) by magnetic fields (MFs). The drug loading was completed by using the hydrophobic interaction of exosome membrane phospholipid bilayer and DOX to obtain exosomes based SPMNs clusters (expressed as D-SMNC-exosomes). After systemic administration, SMNC-exosomes can be retained and accumulated through magnetic interaction with externally applied MFs, thereby increasing the drug concentration at the site of cancer lesions. In order to evaluate the drug release behavior of D-SMNC-exosomes, the in vitro release curves of DOX from SMNC-exosomes were detected at pH 7.4 (physiological environment) and pH 5.0 (late tumor endosomes and lysosomes), respectively. D-SMNC-exosomes released the small amounts of drugs at pH 7.4 (50% within 48 h). At pH 5.0, D-SMNC-exosomes showed relatively rapid and large release (80% within 8 h), followed by continuous and slow release within 2 days, indicating that SMNC-exosomes well protected drugs in blood circulation [93]. In order to evaluate the blood compatibility and histocompatibility of SMNC-exosomes, hemolytic activity test and tissue section analysis of main organs were carried out respectively. The data showed that there were no signs of hemolysis and acute organ damage, indicating that SMNC-exosomes have biocompatibility as a drug delivery carrier [123]. Recently, in the study of subcutaneous tumor model of mouse melanoma, it was found that cell-penetrating peptide (CPP) and TNF-α-Anchored exosomes and superparamagnetic iron oxide nanoparticles (CTNF-α-exosome-SPION) coupling enhanced tumor targeting of TNF-α-exosomes under the action of external MFs. To determine the drug loading distribution of CTNF-α-exosome-SPION, the researchers established the correction curve of bovine serum albumin and TNF-α, and the data results show that CTNF-α-exosome-SPION has a high drug loading rate. Nano drug delivery system with high loading rate can reduce CTNF-α to minimize potential side effects. At the same time, in order to verify the effect of modified exosomes on the pharmacokinetics of drugs, the drug plasma concentration–time curve was designed. Compared with the control group, CTNF-α-exosome-SPION group significantly prolonged the half-life of the drug and increased the action times of TNF-α, which is likely to improve its antitumor properties and reduce doses of TNF-α [124]. The significant improvement in pharmacokinetics of this antitumor drug is likely due to the protective effect of exosomes. The combination of genetic engineering and nano materials has broad prospects in tumor therapy and the application of peptide drugs. It can be seen that in the drug delivery system, the targeting exosomes artificially modified by nano magnetic particles can not only be better extracted and separated under the action of external MFs, but also improve the pharmacokinetics and pharmacodynamics of the therapeutic drugs, so as to enhance the targeting therapeutic effect and reduce adverse toxicity and side effects.

#### Chemical modification

Although the research on chemical modification technology is less explored, the surface modification of exosomes can still be achieved through it. Click chemistry is one of the most commonly used techniques for molecular surface modification [125], which has been used to exploit and prepare targeting nanoparticles [126, 127]. Compared with traditional chemical reactions, click chemistry has many advantages; it can react in aqueous solution in a short time [126], and the conjugation reaction does not affect the size of exosomes and the uptake and absorption of cells, which is suitable for the surface modification of exosomes [128]. Copper-catalyzed azine cycloaddition (click chemistry) is very suitable for conjugation between chemical molecules and biomolecules on the surface of exosomes. It has fast reaction time, high specificity, and compatibility in aqueous buffer. It was found that after the preliminary functional modification of the exosome surface with terminal alkyne, azide fluoride 545 was coupled to the surface functionalized exosome by click chemistry. The size of the exosomes after azide 545 coupling was similar to that of the unmodified exosomes, and azide fluoride 545 enhanced the adhesion/internalization ability of the exosomes to 4T1 cells [128]. After using click chemistry to functionalize the surface of exosomes, we can use markers such as fluorescence, radioactivity, and MRI contrast agents to track exosomes in vivo and observe the biological distribution of exosomes.

Chemical modification can also be achieved by linking large biomolecules to exosomes. In the chemical modification, the ligand/receptor interaction is mainly based on the combination of the over-expressed receptors on the cancer cell surface and the targeting chemicals integrated into exosomal surface, which can realize the targeting aggregation of exosomes to the cancer cell. It is known that CD47 is overexpressed on the surface of most tumors and interacts with signal regulatory protein α (SIRPα) on phagocytes, which greatly limits the ability of macrophages to phagocytize tumor cells. The researchers used transmembrane region structure of platelet-derived growth factor receptors and SIRPα avariant co-modified exosomes (SIRPα-exosomes), which can destroy the interaction of CD47-SIRPαleads to increase the number of cells phagocytized by macrophages, thereby inhibiting tumor growth. In addition, the treatment of SIRPα-exosomes can promote T cell infiltration in syngeneic tumor mouse model and increase the possibility of CD47 targeting therapy to release innate and adaptive antitumor responses. It is worth noting that SIRPα-exosomes can shorten the time required for exosomes to reach the tumor target of CD47 overexpression, and only low-dose SIRPα-exosomes can effectively inhibit the growth of tumor in vivo by blocking CD47-SIRPα, so as to improve the therapeutic effect [129]. The research based on CD47 blocking strategy can effectively antagonize the inhibitory signal transduction mediated by CD47. The use of exogenous antagonists can avoid the potential side effects of anti-CD47 monoclonal antibody treatment, and only a small amount of exogenous antagonists can induce tumor regression in vivo. In conclusion, the establishment of membrane-bound protein surface engineered exosomes by chemical modification provides a good nano immune platform for the treatment of tumors and other diseases.

The insertion of amphiphilic molecules into exosomal lipid bilayers represents another chemical modification strategy. It is known that Sigma receptor is overexpressed in cancer cells [130, 131], including non-small cell lung cancer cells [132], prostate cancer cells [133], and so on. Aminoethyl benzamide is a ligand with high affinity for Sigma receptor [132]. The researchers integrated aminoethyl-anis-amide-polyethylene glycol (AA-PEG) into exosomal membrane to obtain the lung cancer targeting exosomes with Sigma receptor as the target spot, and loaded paclitaxel (PTX) into the targeting exosomes. AA-PEG-exosome carrier loaded with PTX (AA-PEG-exosome-PTX) has high drug loading. After systemic administration, it can accumulate a large amount in tumor cells and prolong the circulation time in blood. In vivo and in vitro experiments showed that AA-PEG-exosome-PTX had higher uptake rate in lung cancer cells than unmodified drug loaded exosomes, had better antitumor effect and prolonged survival time in lung metastasis mice [134]. Surface of EVs, modified by hydrophilic cholesterol coupled with RNA aptamer or folic acid, bind to specific receptors over expressed on cancer cells, and transport siRNA and miRNA to corresponding tumor sites, which enhanced the anti-tumor effect [135]. This study shows that the effective reprogramming of natural EVs using RNA nanotechnology has realized effective cell targeting, siRNA and miRNA delivery, and cancer inhibition, showing the powerful physicochemical properties of reprogrammed EVs, enhanced cancer cell-specific targeting, and effective intracellular release of siRNA to inhibit tumor growth [135]. Another study used biotin and avidin double ligand method to chemically edit the phospholipid membrane of donor cells, and wrapped the drug in the cytoplasm. When the genetically engineered donor cells secrete exosomes, the double ligand and drug can be carried by the exosomes together, and then the exosomes were separated by microfluidic chip method. Compared with no ligand or biotin single ligand modified exosomes, PTX-avidin–biotin-exosomes-treated mice showed longer blood circulation time, well tumor targeting, and more obvious tumor growth inhibition, revealing that double ligand modification can make the targeting ability of exosomes higher in vivo, which further improves the targeting anticancer effect of chemotherapeutic drugs [136]. Double ligand-modified exosomes, with the advantages of long circulation time, low incidence of nonspecific side effects, and good tumor targeting, are the valuable nano material. The combination of targeting and biocompatibility of engineered exosomes provides a powerful and novel drug delivery platform for anti-tumor clinical treatment.

### Comparison of different modification methods

There are many sources of exosome targeting, mainly through the modification of exosome surface proteins to enhance its targeting effect on specific cells and tissues. Homing peptide and targeting receptor protein modification technology is to integrate targeting homing peptide or targeting protein into the surface of exosomes to enhance their tumor targeting. Targeting peptide-modified exosomes not only improve the targeting of drugs, but also promote the vascular and tissue penetration of antitumor drugs [94, 137]. In terms of cardiovascular diseases, a variety of homing peptides have been identified and applied to targeting therapy, including atherosclerosis [138, 139, 140], pulmonary hypertension [141], and myocardial cells with ischemia/reperfusion injury [142]. Regarding the detection of the physiological effects of targeting peptide-modified exosomes on the body, the results showed that no tissue damage and other abnormalities were found in several major organs [95]. At the same time, homing peptide-modified exosomes can recognize early tumor tissues with clear boundaries and shapes [101]. Although more and more studies have successfully designed exosomes as therapeutic drug carriers that can target a variety of receptors, the targeting peptides fused to the N-terminal of exosome membrane protein may also be cut/degraded in the process of transporting the fusion protein, thus losing the targeting [12]. To solve this problem, some studies have found that adding a glycosylated peptide motif GNSTM to the N-terminal of the targeting peptide can effectively protect the homing peptide or targeting receptor protein from protease degradation, increase the expression of the targeting peptide in cells and exosomes, and further enhance the targeting delivery of exosomes to tumor cells [114]. However, whether this method affects the display and/or stability of targeting peptides at the receptor remains to be tested experimentally. At the same time, the possible effects of other components in exosomes and their binding targeting peptides on the recipient immune system need to be further studied. Magnetic nanoparticle modification technology can endow exosomes with superparamagnetism and ferromagnetism at the same time, and can isolate and purify exosomes in blood, which may contribute to the diagnosis of cancer and other diseases [123]. The organic combination of magnetic particles and exosomes is expected to expand its application in biomedical field. The component materials of nano magnetic therapeutic agents are usually selected according to biocompatibility, which may be a complex process, because these materials interact with biological systems, and our current understanding of material biological system interaction is limited [143]. In addition, we should further demonstrate in vivo nanoparticle targeting in large animals to achieve therapeutic effects, because the data of targeting experimental results in rodents may not be reproducible in large animals/humans [143]. Clicking chemical modification on the surface molecules of exosomes will not affect the size of exosomes, and there is no change in the degree of association between exosomes and recipient cells. In addition to affecting the biological distribution of exosomes, click chemistry was also found to be an effective tool for labeling exosomes with fluorescent, radioactive, and MRI reagents to accurately track injected exosomes in vivo [128].

## Discussion

The research of exosomal targeting has gradually become a research hotspot in recent years. With the progress of experimental technology and the improvement of experimental conditions, the increasing numbers of studies have found that exosomes have great potential in clinical diagnosis and treatment. In recent years, some scholars have carried out researches on the targeting of exosomes. This paper mainly reviews the natural targeting of exosome and the strategy and mechanism of optimizing exosomal targeting by modification, and its potential value in clinical application was discussed.

As a natural drug carrier, exosomes can correspondingly improve the pharmacokinetic effects such as drug solubility, stability, and bioavailability, so as to reduce the side effects caused by the non-specific distribution of the drug. Because of their surface proteins and other components, exosomes have a certain natural targeting, which can be used as drug carriers in the application of disease immunotherapy to enrich specifically to target cells or tissues and realize the targeting transportation of goods. Compared with traditional direct drug therapy, drug packed in exosome with targeting delivery can reduce drug toxicity and enhance therapeutic efficacy to a certain extent. In addition, we can optimize exosomal targeting by surface modification. The targeting modification of exosomes is carried out by the engineering modification technology to enhance the enrichment ability of the exosome in the specific part and its affinity to the target cell or target organ. By packing the drug into the modified exosomes, more special cell or organ targeting delivery of the drug can be realized, which makes exosomes as the targeted carriers to play a better effect in clinical treatment. The ligands or peptides were integrated into exosomal surface by engineering targeting modification, and the specific recognition between ligands and receptors was used to enhance the targeting of the exosome in the recipient cells. With the in-depth study of exosomal targeting, therapeutic systems such as organ targeting drug delivery system and exosome-mediated targeting signal transduction are expected to provide new ideas and methods for the treatment of diseases.

As a nanoparticle carrier for drug delivery, exosomes still face different challenges in the clinical treatment of diseases. In recent years, the effects of nano protein interaction and the formation of protein corona (PC) on the transport fate of nano carriers have been confirmed [144, 145]. The formation of protein corona is affected by many factors, mainly including the physical and chemical properties of nanoparticles and nanoparticles (NPs) exposure concentration. It is found that the traditional oral drug treatment results for inflammatory bowel disease, colon cancer, and other colon diseases are not ideal, but after NPs enzyme corona complex is transported to the colon, after bacteria degrade enzymes, canopy and nanoparticles, its loaded drugs can be released in the colon to achieve targeted treatment of colon diseases [146]. Researchers found that although the targeting of NPs can be promoted by modifying the surface, the results are often disappointing. Once injected into the physiological environment, NPs will interact with protein biological components and be wrapped by PC [147, 148]. This reaction can trigger the immune response and affect the toxicity and targeting ability of NPs [149, 150]. This can lead us to think about the effects of nano protein interaction and protein corona on exosomal transmission. The issue remains to be studied, which is whether exosomal surface proteins interact with proteins or form protein corona and affect the drug delivery of exosomes in the immune system. In addition, the targeting transformation of exosomes still needs to overcome some limitations: including how to avoid non-specific targeting, how to give the exosome targeting ability without destroying the exosomal structure and content, and so on. Then, whether the modified exosomes can maintain the natural basic characteristics such as low immunogenicity, etc., which needs to be further studied. In a word, exosomes can be modified to become better targeting delivery carriers in clinical application, which has a broad application prospect, but it still needs in-depth research and exploration by researchers. Therefore, we hope to have a better exosomal modification method to overcome the limitations of the current strategy. As these issues might be some of the main reasons, limiting the value of these targeting exosomes for clinical applications. If the above problems can be solved, exosomes will become the important carriers for clinical loading of chemotherapeutic drugs in the treatment of tumor, and have a good prospect of clinical application.

Exosomes as the carrier have many advantages, but also have some shortcomings and defect. The separation and purification of exosomes is one of the bottlenecks in basic research and clinical application of exosomes. The exosomes secreted by cells change with the changes of cell type and physiological state, and the differences of separation methods will also affect the types of exosomes. The output of naturally produced exosomes is generally small, which is difficult to produce in large quantities, and there is no unified gold standard for the extraction and purification method of exosomes, which limits the output of exosomes from various body fluids. Due to many factors, such as small volume, complex components, low yield of separation and purification, and difficult to control by engineering modification technology, it is not very sure whether the obtained engineering exosomes can be used in clinical disease diagnosis and treatment. Therefore, it is still difficult for exosomes to be more widely used in clinic. At present, there are few studies on the quantitative or qualitative analysis of the targeting of exosomes in cells or organs. In addition, exosomes have complex heterogeneity, and the detailed mechanism by which the biogenesis, cell origin, and biomolecular composition of exosomes affect pharmacokinetics is unclear. In order to speed up the application of exosomes as targeting delivery carriers, it is necessary to develop and standardize schemes for obtaining high-quality and high-purity targeting exosomes, as well as techniques for effectively loading therapeutic drugs into exosomes. Therefore, more in-depth research is needed to use exosomes as targeting carriers for disease treatment, especially the mechanism of exosomes targeting cells or organs, which will be helpful to tap the potential of exosomal targeting in clinical application.

## Conclusions

In recent years, limited breakthroughs have been made in the use of exosomes as drug carriers in the treatment of diseases. Exosomes are natural carriers of biomacromolecules, which make them attractive candidates for delivering therapeutic biomolecule. There are still some challenges in the field of exosomes using their natural targeting for drug delivery. Before exosomes have made a great leap forward as the drug delivery system, the optimization of exosomal targeting is helpful to solve the problem of targeting drug delivery and to develop an effective treatment plan for the disease. In this review, we introduce the natural targeting of exosomes, and summarize the related research on the engineering modification methods to optimize exosomal targeting. Methods such as modifying targeting peptides or targeting proteins to the surface of exosomes and the physical or chemical modification can be used to enhance the ability of exosomes to target cells or organs and improve the efficiency of targeting delivery, so as to provide new ideas and strategies for targeting treatment of diseases and bring new opportunities and challenges for the application of exosome targeting in clinical treatment.

## Data Availability

Not applicable.

## Abbreviations

EVs: Extracellular vesicle
MVBs: Multivesicular bodies
ILVs: Intracellular vesicles
siRNA: Small interfering RNA
miRNA: MicroRNAs
ESCRT: Endosomal sorting complex required for transport
mRNA: Messenger RNA
lncRNA: Long non-coding RNA
circRNA: Circular RNA
GPI: Glucose phosphate isomerase
SIRPα: Signal regulatory protein alpha
MFG: Milk fat globule
Cur: Curcumin
Lamp2b: Lysome-associated membrane glycoprotein 2B
imDCs: Immature dendritic cells
Dox: Doxorubicin
IMTP: Ischemic myocardial targeting peptide
cRGD-Exo: C(RGDyK)-coupled exosomes
SPIONs: Superparamagnetic iron oxide nanoparticles
Met: Mesenchymal-epithelial transforming
TNBC: Triple-negative breast cancer
EGFR: Epidermal growth factor receptor
RVG: Rabies viral glycoprotein
BBB: Blood-brain barrier
Apo-A1: Apolipoprotein Amur1
SR-B1: Scavenger receptor B type 1
IL3-R: Interleukin-3 receptor
CML: Chronic myeloid leukemia
HEK: Human embryonic kidney
RTC: Reticulocyte
TfR: Transferrin receptors
SPMNs: Superparamagnetic nanoparticles
SMNC: Superparamagnetic nanoparticle cluster
MFs: Magnetic fields
CPP: Cell-penetrating peptide
SIRPα: Signal regulatory protein α
AA-PEG: Aminoethyl-anis-amide-polyethylene glycol
PTX: Paclitaxel
PC: Protein corona
NPs: Nanoparticles
